# Annual Convocation of Niagara University

**Published:** 1888-04

**Authors:** 


### *ANNUAL CONVOCATION OF NIAGARA UNIVERSITY.*

The annual convocation and meeting of the alumni of the Medical Department of Niagara University will take place in this city on April 10th. The exercises will take place in the morning, afternoon and evening. The morning session will be at the college building, and commence at 11 A. M. Professor Thomas Lothrop will deliver the address of welcome, which will be followed by the address of the president, Professor Floyd S. Crego. Immediately thereafter, the reception of new members will occur, which is to be followed by the election of officers of the association. The afternoon session will be of a medical character entirely, and, as will be seen by the programme, the committee have obtained men of prominence to take part in the afternoon session. It is as follows: Some of the Relations of Pathology to the Practice of Medicine and Surgery, by Frank W. Ross, M. D., Elmira, N. Y.; The Diagnosis of Contracted or Granular Kidney, by A. D. Lake, Perrysburgh, N. Y.; An Inquiry into the Importance of Physical Culture as a Branch of Preventive Medicine, by William C. Bailey, M. D., Albion, N. Y.; A Detailed Account of the Weir Mitchell Treatment, by Edward B. Angell, M. D., Rochester, N. Y. The convocation exercises will be held in the evening at Association Hall, Y. M. C. A. building. The address to the graduates will be made by Henry Flood, M. D., of Elmira, N. Y., this to be followed by a general address by Rev. J. T. Edwards, D. D., president of the Chamberlain Institute. The profession generally are invited to attend each of these exercises. The banquet, which follows the exercises, will be held at the Genesee hotel, and will be attended by members of the Alumni Association and their invited guests.

